# High Glass Transition Epoxy Resins from Biobased Phloroglucinol and Unmodified Kraft Lignin

**DOI:** 10.1021/acsomega.5c06542

**Published:** 2025-11-04

**Authors:** Jan Janesch, Roxana Dinu, Thomas Rosenau, Antje Potthast, Wolfgang Gindl-Altmutter, Stefan Grasböck, Irina Sulaeva, Alice Mija

**Affiliations:** † BOKU University, Institute of Wood Technology and Renewable Materials, Department of Natural Sciences and Sustainable Resources, Konrad-Lorenz-Straße 24, 3430 Tulln, Austria; ‡ BOKU University, Institute of Chemistry of Renewable Resources, Department of Natural Sciences and Sustainable Resources, Konrad-Lorenz-Straße 24, 3430 Tulln, Austria; § Wood K Plus—Competence Centre for Wood Composites & Wood Chemistry, Kompetenzzentrum Holz GmbH, Altenberger Straße 69, 4040 Linz, Austria; ∥ BOKU University, Core Facility Analysis of Lignocellulosics (ALICE), Konrad Lorenz-Strasse 24, 3430 Tulln, Austria; ⊥ 27046 Côte d’Azur University, Institute of Chemistry of Nice, UMR CNRS 7272, 06108 Nice Cedex 2, France

## Abstract

This study investigates the development of high-performance epoxy resins derived from potentially biobased phloroglucinol triglycidyl ether (TGPh) and unmodified Kraft lignin (KL), aiming to create thermoset materials from renewable resources. Two types of KL, sourced from hardwood and softwood, were incorporated into the TGPh matrix at high loadings of 30% wt. to achieve materials with potentially ∼95% renewable content. The resulting epoxy resins were characterized using thermally resolved Fourier-transform infrared spectroscopy (FTIR), differential scanning calorimetry (DSC), thermogravimetric analysis (TGA), dynamic mechanical analysis (DMA), tensile testing, and scanning electron microscopy (SEM). All samples exhibited >97% gel content after 3 days in various solvents, indicating complete cross-linking. DSC and FTIR analyses confirmed the interaction of KL with the resin, suggesting that lignin may act as a cohardener. The addition of lignin increased the glass transition values (*T*_g_) from 146 °C for the pure TGPh homopolymer to 178 and 187 °C for TGPh cured with softwood and hardwood KLs, respectively, indicating an increased cross-link density. Although the tensile strength of the resins decreased from 51.1 ± 16.0 MPa (mean ± SD) for the TGPh homopolymer to approximately 20.0 ± 2.8 MPa (mean ± SD) and 16.3 ± 2.3 MPa (mean ± SD) for the TGPh thermosets obtained with softwood and hardwood KLs, respectively, the stiffness was maintained at a tensile modulus of 2–2.5 GPa. SEM analysis revealed inhomogeneities in the lignin-containing samples, potentially explaining their lower mechanical properties. The findings demonstrate the potential of these biobased epoxy resins, particularly for applications such as electronics, automotive, and aerospace, which require high glass transition temperatures. Moreover, the results help in understanding the active action of lignin in the cross-linking of epoxy resins.

## Graphical abstract

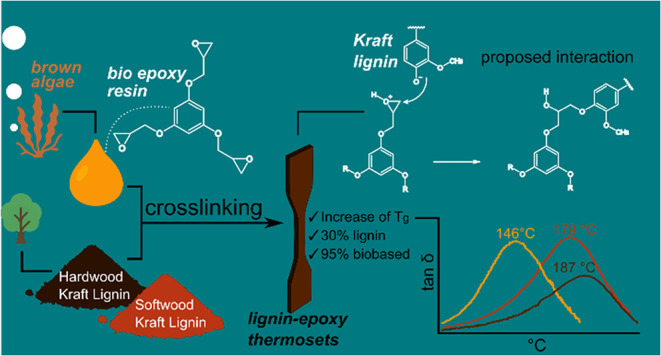

## Introduction

Epoxy resins are crucial for a variety of applications due to their durability, rigidity, solvent resistance, high glass transition values, and excellent mechanical properties. Traditionally, these resins are fossil-based and often contain compounds derived from bisphenol A, which pose toxicological risks.^1^ Consequently, over the past few decades, significant research efforts have been directed toward developing epoxy resins from biobased and nontoxic alternatives.

One of the most promising biobased resources for the preparation of epoxy resins is lignin. Lignin is a phenolic biopolymer that constitutes about 15–40% of wood, making it the most abundant aromatic polymer on Earth.^2^ Its aromatic cross-linked structure is optimized by nature to provide stiffness to trees. It is thus no surprise that lignin lends itself to applications in thermosetting resins. Specifically, the fact that the addition of lignin may enhance the glass transition values of polymers is an often-named argument for its use.^3^

The pulp and paper industry produces technical lignin as a byproduct in enormous quantities, most of which is currently incinerated for energetic use.^4^ The Kraft process is the dominant pulping process, accounting for more than three-quarters of the produced pulp.^5,6^ One study has identified the annual global production of KL to be 265 million tons in 2018.^7^ From the perspectives of sustainability, circularity, and economics, there is significant interest in converting KL into value-added materials, with its use in epoxy resins being a promising route.^4,8^

In the Kraft process, a multitude of reactions take place, resulting in a lignin which greatly differs from the structure of native lignin in wood.^2^ Phenolic OH groups, aliphatic OH groups, as well as minor amounts of COOH groups, are the dominant functional groups in KL^9,10^ and offer potential toward its modification.

One of the most explored uses of KL in thermoset resins is its conversion into epoxy resins by reaction of lignin’s hydroxyl groups with epichlorohydrin. The resulting glycidylated lignin has been used in several studies as an alternative for bisphenol A diglycidyl ether (DGEBA), the most common base epoxy in epoxy resin.^8,11^ This route is intensively investigated and is well-documented in the literature.^8,11–13^

In addition to glycidylation, some studies have explored the potential role of lignin in the curing of epoxy resins. In this context, most studies follow the modification of lignin, usually with the aim to increase its nucleophilic character and thus its reactivity toward epoxy ring-opening reactions. Various modification methods have been described in this context, including carboxylation by ozone treatment,^14,15^ treatment with anhydrides,^14,16–18^ phenolation,^19,20^ demethylation,^20^ and carbonation.^21^

However, these modifications often require multiple synthesis steps involving toxic, volatile, and expensive compounds. Despite the often-claimed low reactivity of lignin, several studies have investigated the addition of unmodified lignin to epoxy resins, and indicated that lignin actively participates in the curing reaction.^11,22–25^ This interaction has been confirmed by presence of exothermic curing peaks in differential scanning calorimetry and a consumption of epoxy groups in FTIR.^23–26^ In one study, lignin has also been used as the sole cross-linking agent of an epoxy resin, achieving cross-linking without the involvement of other hardeners.^27^ In the literature, lignin’s role in epoxy resins has been described as one of cross-linking filler,^11^ catalyst,^23^ curing agent,^23^ or reactive additive^24^ when reacted with epoxy resins at high temperature, typically without additional catalysts. It is generally believed that lignin’s phenolic OH groups participate in epoxy ring opening, although aliphatic OH groups may as well play a role.^22,23^ Moreover, phenolic hydroxyl groups may act as proton donors, catalyzing epoxy ring opening reactions.^23^

Although some articles have reported negative effects of adding unmodified lignin to epoxy resins, particularly concerning tensile strength and brittleness,^22,28^ some results also highlight the beneficial impact of lignin as a (co)­hardener on the properties of epoxy resins. In several instances, the addition of lignin to epoxy resins has been associated with increased glass transition values and higher cross-linking densities.^3,22,23,28^

Many studies on this topic have incorporated lignin into a petroleum-based epoxy resin, typically DGEBA, which is the most common monomer used for formulating epoxy resins. Unfortunately, DGEBA has been associated with several health issues and environmental concerns.^1^ This has driven the search for safer, biobased alternatives. Therefore, in recent years, several epoxy resins from different renewable sources have been investigated..^22,29–34^ However, only a couple of studies have described such bioderived epoxy resins in combination with lignin as (co)­hardener.^22,28,30^

The triglycidyl ether of phloroglucinol (TGPh) is an aromatic epoxy monomer that can be synthesized from biobased compounds and has been described in recent literature.^28,32,35–38^ Specifically, TGPh can be obtained by the glycidylation of phloroglucinol, a biobased compound belonging to the group of phlorotannins found in brown algae.^39^ TGPh is characterized by its three epoxy groups per molecule (Scheme 1). It has been shown that epoxy resins prepared from TGPh had higher reactivity, as well as higher cross-linking density and glass transition temperatures than resins prepared from DGEBA, which has been attributed to TGPh’s trifunctionality.^35,37^ In past studies, TGPh has been cross-linked with amines,^32,35,37^ as well as biobased anhydrides^38^ and humins.^36^

**Scheme 1:**
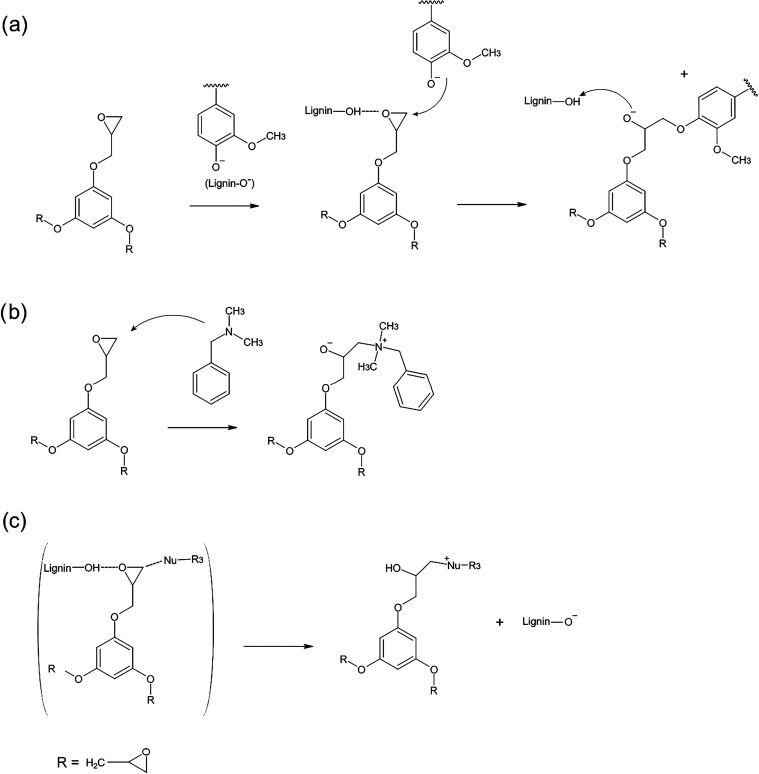
Potential Reactions Taking Place in the Curing of TGPh-Based Epoxy Resins: (a) Curing with Lignin; (b) with Tertiary Amine, (c) Curing Proceeding through Trimolecular Transition State Stabilized by Phenolic Hydroxy Groups

Herein, we hypothesized that adding KL as a hardener to TGPh may lead to biobased thermosets with improved thermomechanical properties. Two types of KL, one from hardwood and one from softwood, were used at high loadings of 30% wt. to achieve materials with potentially ∼95% renewable content. The performance of the materials was compared to thermosets prepared by TGPh’s homopolymerization.

## Experimental Section

### Materials

Toluene (≥99.5%, CAS 108–88–3), Acetone (≥99.5%, CAS 67–64–1), 2,4,6-Tris­(dimethylaminomethyl)­phenol (DMP-30, ≥95%, CAS 90–72–2), *N*,*N*-dimethylbenzylamine (BDMA, ≥99%, CAS 103–83–3), and bisphenol A diglycidyl ether resin (DGEBA, CAS 1675–54–3) were all obtained from Sigma-Aldrich. Tetrahydrofuran (>99.5%, stabilized, CAS 109–99–9) was obtained from Carl Roth GmbH.

TGPh was synthesized via a glycidylation reaction of phloroglucinol with epichlorohydrin under basic conditions, following protocols from the literature and patented procedures.^40–42^ In a typical reaction, phloroglucinol was dissolved in epichlorohydrin and stirred under reflux. Aqueous sodium hydroxide was added dropwise to initiate the reaction, promoting the formation of glycidyl ethers. After completion, the reaction mixture was neutralized, washed, and purified by vacuum distillation and solvent extraction to remove unreacted reagents and byproducts. TGPh appeared as a yellowish viscous resin at ambient laboratory temperature (estimated 20–25 °C), had an average Mw of 295 g/mol, EEW of 115 g/mol, and functionality of 2.56 epoxy groups per molecule.

### Kraft Lignin (KL)

The two types of Kraft lignins used in this study—a hardwood KL (Suzano, Brazil), and a softwood KL (Indulin AT, MeadWestvaco, USA)—were obtained in powdered form. Functional group content of the KL was determined by ^31^P NMR as described in ref 43. Molar mass distribution was measured using size exclusion chromatography in accordance with the literature.^44^ In brief, lignin samples and reference standards were dissolved in DMSO/LiBr (0.5% w/v) at a concentration of 10 mg mL^–1^, shaken overnight, and subsequently filtered through a 0.45 μm PTFE membrane prior to analysis. For PSS standards, dissolution in DMSO was facilitated by a cation-exchange resin pretreatment, as reported in the literature. Results are presented in the SI (Figure S1 and Tables S1 and S2). KL was dried in a vacuum oven for 24 h at 50 °C, stored in a desiccator, and used without further purification.

### Sample Preparation

The thermosets were developed starting from the laboratory-synthesized TGPh. Thermosetting resins were prepared by mixing TGPh with an accelerator to prepare TGPh homopolymer. Mixtures of TGPh with lignin were prepared at 30% wt. lignin, the highest loading that still allowed a workable viscosity. A 5% wt. initiator/accelerator mixture, consisting of 2.5% wt. *N*,*N*-dimethylbenzylamine (BDMA) and 2.5% wt. 2,4,6-Tris­(dimethylaminomethyl)­phenol (DMP-30), was used in the experiments and is in the following text referred to as *accelerator*.

Table 1 provides an overview of the ratios of components in the samples used in this study.

**Table 1:** Sample Code and Ratios of Samples Prepared Using Triglycidylether of Phloroglucinol (T), Softwood Kraft Lignin (SKL), Hardwood Kraft Lignin (HKL), and Accelerator (A)

sample name	TGPh (% wt.)	softwood Kraft lignin (% wt.)	hardwood Kraft lignin (% wt.)	accelerator (% wt.)
T-A	95	-	-	5
T-SKL-A	65	30	-	5
T-HKL-A	65	-	30	5
T-SKL	70	30	-	-
T-HKL	70	-	30	-

Sample preparation was performed in accordance with a previously described procedure.^28^ First, TGPh was briefly warmed in a 40 °C water bath to reduce its viscosity. To prepare sample T-A, the accelerator was hand-mixed into TGPh. For the preparation of T-SKL-A and T-HKL-A, the respective amount of lignin was mixed into TGPh, and the mixture was put into an ultrasonic bath at 40 °C for 10 min to allow for good mixing of the components. The mixture was left to cool to ambient laboratory temperature (estimated 20–25 °C), and 5% wt. of accelerator mixture (2.5% wt. BDMA, 2.5% wt. DMP-30) was hand-mixed into the resin. The prepared mixtures were poured into silicone molds of desired shape and cured in a convection oven at 70 °C, 90 °C, 120 °C, and 200 °C, for 1 h at each temperature. Samples T-SKL and T-HKL were prepared from TGPh and KLs without the addition of accelerator. These samples were only used in the FTIR curing study.

### Characterization

#### Attenuated Total Reflection Fourier-Transform Infrared Spectroscopy (ATR-FTIR)

Spectra of the raw materials and the materials after oven-curing were obtained using a NICOLET iS50 FTIR (Thermo Scientific) in the spectral range of 400 cm^–1^ to 4000 cm^–1^, with 32 scans per spectrum at a resolution of 2 cm^–1^.

To study the reactivity of the epoxy systems, fresh reaction mixtures (as detailed in Table 1) were subjected to temperature-modulated ATR FTIR using a PIKE Technologies GladiATR heating plate. The heating rate was set to 5 K/min, and spectra were recorded every 10 °C from 40 to 200 °C. Fresh reaction mixtures were prepared as described above, but without the use of an ultrasonic bath.

All FTIR spectra were processed using Spectragryph software. The baseline was set to 1540 cm^–1^, and the area from 1540 cm^–1^ to 1675 cm^–1^, which corresponds to the aromatic band of TGPh and KL, was normalized.

#### Thermal Analysis

Differential Scanning Calorimetry (DSC) was carried out on a DSC 3 STAR^e^ System (Mettler Toledo) operated by the manufacturer’s STAR^e^ Software. To obtain the curing profile and enthalpy, fresh samples were heated from 25 to 250 °C at a heating rate of 10 K/min in closed steel pans.

Thermogravimetric analysis (TGA) was carried out in a TGA 2 STAR^e^ System (Mettler Toledo) at 10 K/min from 25 to 1000 °C under 50 mL/min airflow.

Dynamic Mechanical Analysis (DMA) was carried out using a DMA 1 STAR^e^ System (Mettler Toledo). Samples with approximate dimensions of 50 × 7 × 2 mm^3^ were tested in three-point bending mode over a temperature range of −150 to 275 °C, at a heating rate of 3 K/min, a frequency of 1.0 Hz, and an amplitude of 20 μm.

#### Mechanical Testing

Shore hardness was measured using a Zwick Roell 3116 hardness tester on five cured samples per variant. Each sample was tested seven times, and the mean and standard deviation were calculated.

Tensile testing was carried out using an Instron 34SC-5 universal testing machine with a 5 kN load cell. Dogbone-shape samples with a gauge length of 35 mm and width of ∼5 mm were sanded to obtain an even and defect-free surface. Five samples per formulation were tested in tensile mode using a crosshead speed of 5 mm/min. The mean and the standard deviation were reported.

#### Determination of the Gel Content (GC %)

Samples of approximate dimensions of 8 × 5 × 1.5 mm^3^ were cut from cured specimens, and their initial oven-dry mass (*m*_i_) was determined after drying for 6 h at 50 °C in a vacuum oven. Samples were separately immersed in 3.8 mL toluene, acetone, tetrahydrofuran, or water for 3 days, then dried at 50 °C for 24 h in a convection oven. The final oven-dry mass (*m*_f_) was determined, and the gel content was calculated by eq 1.

1 $$\mathrm{GC}\;\left(\%\right)=\frac{m_{f}}{m_{i}}\cdot 100\%$$

#### Scanning Electron Microscopy (SEM) Imaging

Fracture surfaces were imaged using a TM3030 tabletop scanning electron microscope (Hitachi). Before imaging, a 10 nm thick layer of platinum was coated onto the surfaces using an EM ACE200 sputter coater (Leica). The imaging was carried out at an acceleration voltage of 15 kV.

## Results and Discussion

### TGPh-Lignin Reactivity Evaluated by Fourier-Transform Infrared (FTIR) Spectroscopy Analysis

The study began with an investigation of the curing behavior of the epoxy resin and the effect of lignin on the curing reactions. The curing process is most effectively analyzed by focusing on the spectral region related to epoxy rings (Figure 2). Characteristic bands are identified at 860 cm^–1^, corresponding to the asymmetric C–O–C stretch (830 cm^–1^ for DGEBA^45^), the vibration at 906 cm^–1^ (915 cm^–1^ for DGEBA^45,46^ ), and 765 cm^–1^.^36^ These bands were either absent or had strongly decreased in intensity when spectra were recorded after curing at 200 °C, indicating a curing reaction involving the epoxy groups. This was true for samples with and without an accelerator. Another indicator for curing involves the area at 1060–1180 cm^–1^, related to the various C–O stretching vibrations.^45^ Upon curing, this area experiences a transformation as illustrated by a comparison of T-HKL-A before and after curing in Figure 1. Curing causes a broadening of the signals, indicating the formation of C–O bonds present in a broad range of different chemical environments. It is thus indicative for the formation of a cross-linked polymer material.

**Figure 1:**
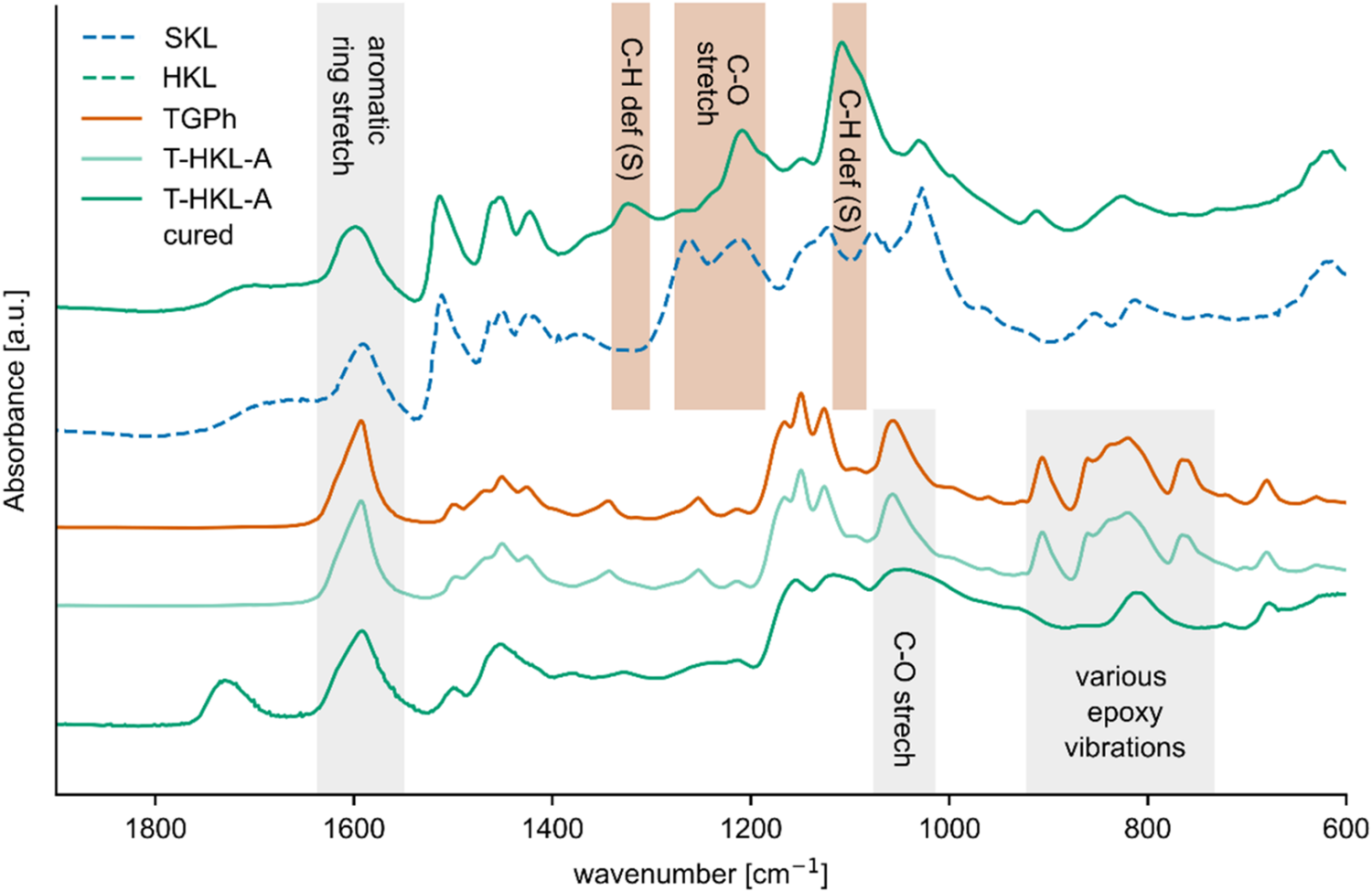
Characteristic bands of pure Kraft lignins (HKL, SKL), uncured TGPh, and uncured and cured mixtures of TGPh and HKL with accelerator (T-HKL-A).

To get more insight into the curing kinetics of mixtures with and without catalyst, the evolution of the epoxy peak at 906 cm^–1^ upon curing was studied in more detail. This band is typically used to study the conversion of epoxy resins.^46^ For this purpose, dynamic ATR-FTIR was conducted by heating samples to 200 °C and recording spectra at every 10 °C (Figures 2 and S3). In all studied samples, heating resulted in a gradual decrease in the intensity of the epoxy band, indicating the occurrence of epoxy-ring-opening reactions. In TGPh without catalyst, a slow intensity decrease could be observed (Figure 2a). When lignin was added, the intensity reduction was increased compared to pure TGPh (Figures 2b and S3a). Moreover, the slope of this reduction was steeper. Both observations indicate the active action of lignin in the resin, contributing to epoxy conversion. The addition of accelerator into the mixtures led to a strongly decreased or even absent band at 906 cm^–1^ for both lignin types (Figures 2c and S3b). The conversion of epoxy groups (α) was calculated by integrating the band between 877 and 920 cm^–1^, and the results are given in Figure 2d. In the absence of accelerator, the TGPh monomer was partially converted with an ultimate conversion α ∼ 0.3. The comparably slow reaction likely progresses through the action of aliphatic OH groups in TGPh engaging in epoxy ring opening reactions. Addition of accelerator to TGPh (T-A) led to faster conversion and reached conversions of α ∼ 0.73.

**Figure 2:**
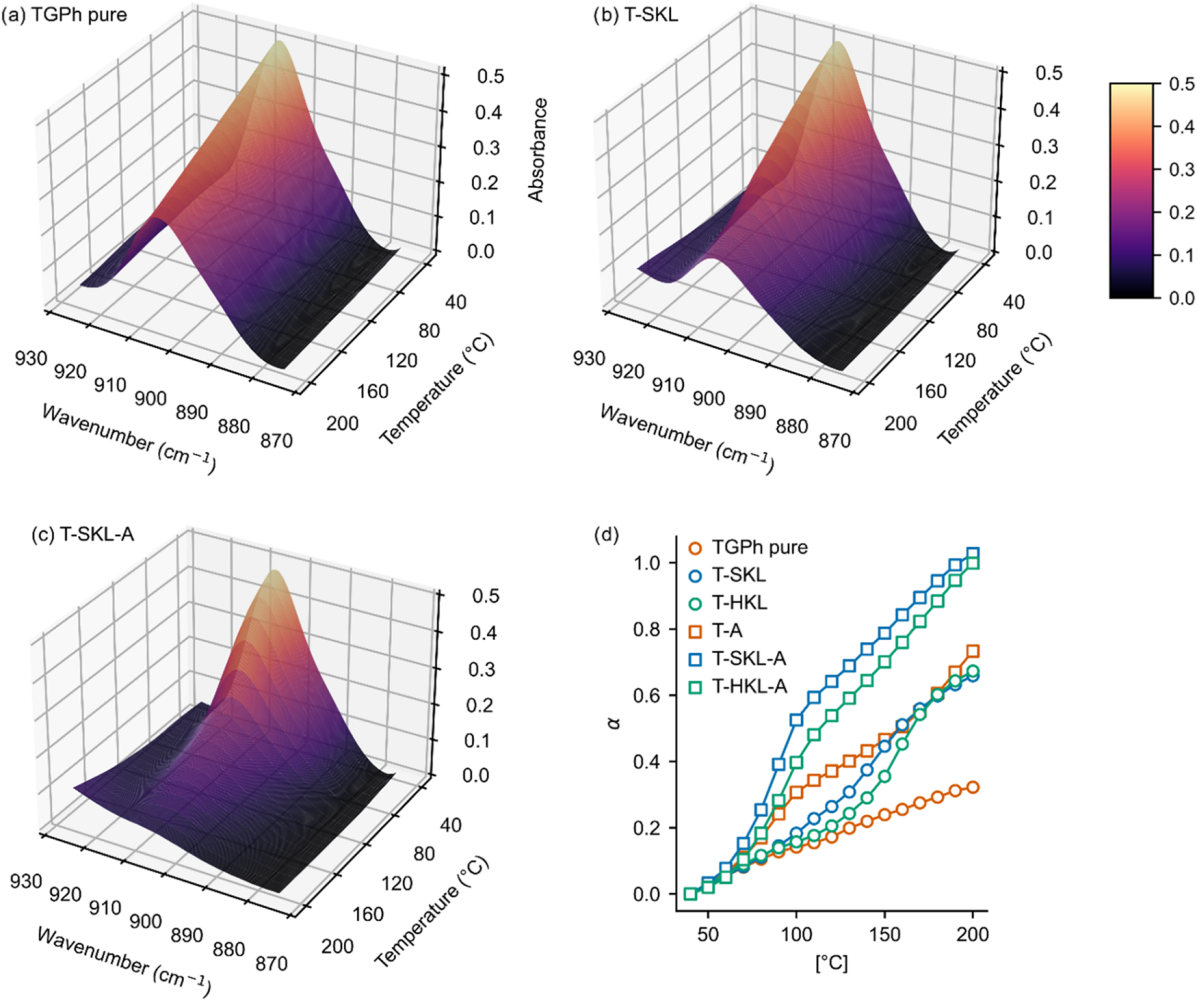
Evolution of the absorption peak intensity of the epoxy vibration band at 906 cm^–1^ in thermally resolved ATR-FTIR for TGPh without accelerator (a), T-SKL (b), and T-SKL-A (c); conversion α calculated from integration of the band at 906 cm^–1^ (d).

Mixtures of TGPh with Kraft lignin (T-SKL, T-HKL) in the absence of accelerator led to higher conversion, which is in a similar range as T-A, and is characterized by a steeper slope and higher conversion compared to TGPh monomer without accelerator.

Lastly, systems containing TGPh, lignin, and accelerator were studied. These systems led to a full conversion of the epoxy functions within the time and temperature range and are characterized by steeper slopes than the other systems.

We note that, for HKL, conversion values from this method are less accurate than for SKL because an overlapping HKL band at 912 cm^–1^ may contribute to the integrated area (Figure S4).

The sigmoidal shape of the conversion curves for mixtures containing lignin suggests an autocatalytic effect, which was absent in the curve of TGPh alone. First, the addition of lignin provides numerous hydroxyl groups. Second, the presence of the more acidic phenolic OH groups in the lignin may be a source of phenoxide, a strong nucleophile which can open epoxy rings, resulting in the formation of ether linkages and new OH groups, thus further contributing to the autocatalytic effect. Lignin’s role in curing epoxy resins has been discussed in literature.^11,22–24^ The phenolic OH groups have been described to be more reactive toward the attack of the epoxy ring (Scheme 1a), but aliphatic OH groups can also participate in the curing under certain conditions.^22,23,47^

Although detailed mechanistic studies focusing on lignin’s OH groups are missing, density functional theory calculations on the reaction of epoxy resins with phenol have been reported.^48^ The authors presented an activation energy of ∼130 kJ/mol for the isolated reaction of the epoxy ring and the phenol’s OH group.^48^ In another pathway, in which a hydroxyl group from a second phenol hydrogen-bonds to the epoxy oxygen, the activation energy was found to decrease to <49 kJ/mol.^48^ This is referred to as the self-promoting pathway, as the presence of phenolic OH promotes the curing.^48^

Such a mechanism, where phenolic hydroxyl groups may act as proton donors, further catalyzing epoxy ring-opening reactions, has also been suggested for lignin (Scheme 1a).^23^ Our results align with these findings, showing that lignin’s phenolic OH groups significantly contribute to the epoxy ring-opening reactions. Both hardwood Kraft lignin (HKL) and softwood Kraft lignin (SKL) demonstrated comparable reactivity in the curing process. The earlier rise in the conversion curves T-SKL and T-SKL-A suggests a slightly increased reactivity for samples containing SKL over samples containing HKL.

The accelerators BDMA and DMP-30 are Lewis bases and have been described as anionic initiators.^49^ They have been used in previous studies for homopolymerization of epoxy resins as well as their cross-linking with other curing agents. While the exact action of these amines is not yet fully understood, several routes are postulated.^48,49^ In the first mechanism, the initiators can directly undergo nucleophilic attack of the epoxy carbon, forming an alkoxide ion, which continues the anionic polymerization (Scheme 1b). Moreover, curing may proceed through a trimolecular transition state, requiring a hydrogen donor (Scheme 1c). It is believed that alkanolamine-type accelerators such as DMP-30 work through this mechanism.^49,50^ Similar to the self-promoted pathway above, phenolic OH groups may stabilize this transition state through hydrogen bonding, thereby aiding the curing process (Scheme 1c).^48^ A third described pathway is the formation of hydrogen-bonded species from tertiary amines and phenolic OH.^48^

To summarize, lignin functions both as a nucleophile (Scheme 1a) and as a hydrogen donor in the self-promoting pathway, demonstrating a dual role as both hardener and accelerator.

### TGPh-Lignin Reactivity Evaluated by Differential Scanning Calorimetry

Differential scanning calorimetry was carried out to get further insight into the curing of the epoxy resins (Figure 3). Pure TGPh exhibited no exothermic peak when heating to 250 °C. Heating TGPh with SKL or HKL without an accelerator showed the presence of broad and low-intensity exothermic peaks, confirming the cross-linking reactions already suggested by the FTIR measurements. The reaction started at elevated temperatures >140 °C and peaked at 160–180 °C. The peak for SKL appeared at slightly lower temperatures than that for HKL, matching the apparent increased reactivity for SKL observed in FTIR (Figure 2d). Moreover, the normalized curing enthalpies were in the range of only 60–80 J/g, suggesting that the presence of lignin without amine accelerators may not lead to effective curing, at least in the observed temperature range.

**Figure 3:**
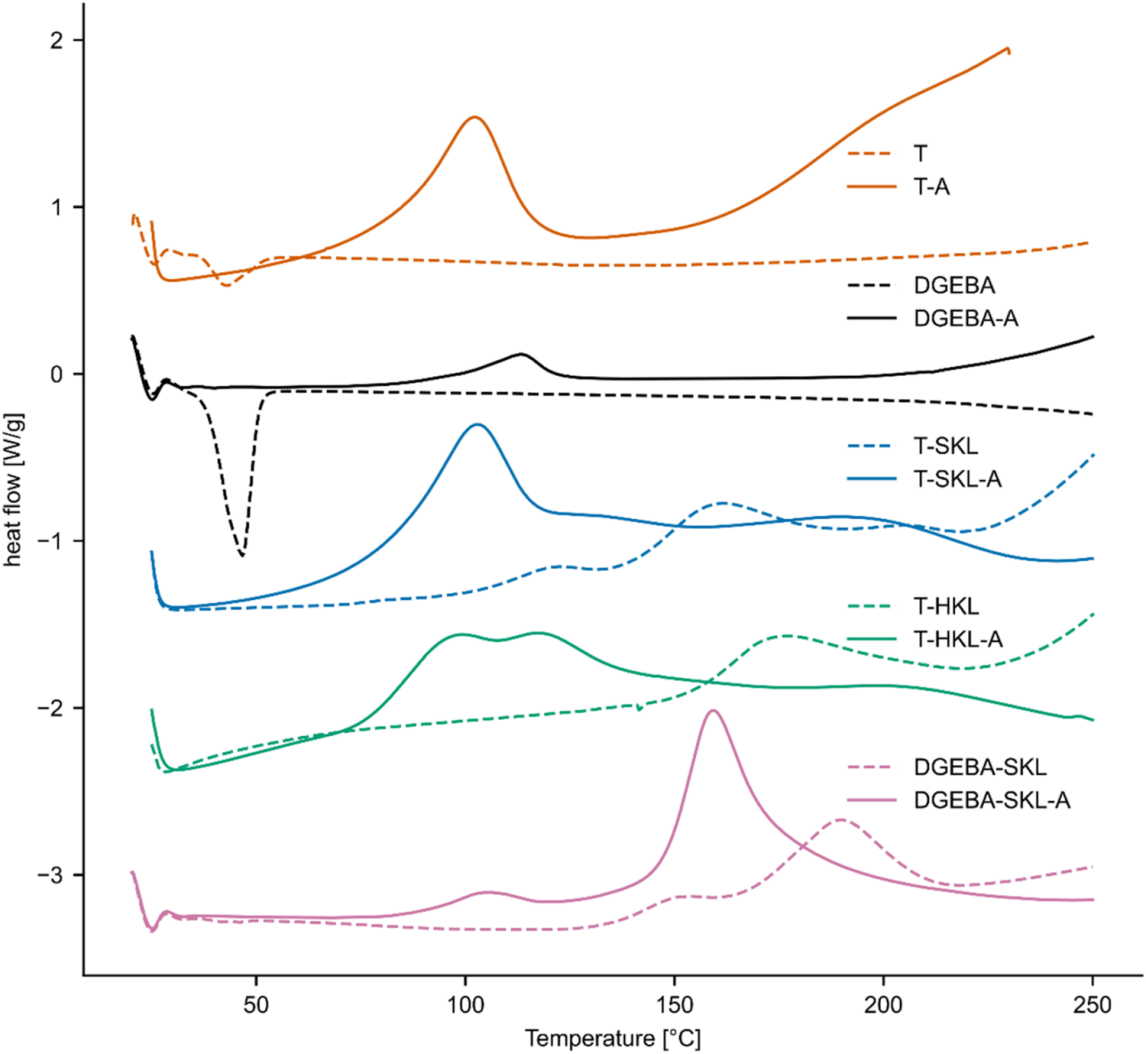
Differential scanning calorimetry (DSC) heat-flow curves for TGPh (T) and bisphenol A diglycidyl ether (DGEBA), and for their blends with softwood (SKL) and hardwood (HKL) Kraft lignins. Dashed lines denote samples without catalyst; solid lines denote samples containing catalyst (suffix “-A”).

In the presence of the accelerator, the polymerization reactions shifted to lower temperatures, with peaks ∼100 °C and onsets around 80 °C. Moreover, curing enthalpies were greatly increased with respect to systems without accelerator. In fact, addition of the accelerator to the mixture of TGPh and HKL, led to the gelation of the resin system at ambient laboratory temperature (estimated 20–25 °C) within minutes. We attribute this rapid gelation to the high reactivity of the resin system, which likely caused the measured enthalpy to vary greatly. The normalized enthalpies were in the range of 140–240 J/g for T-HKL-A and T-SKL-A.

Mixing of TGPh with accelerator (T-A) showed a single prominent peak, with an onset temperature and a peak maximum of about 80 and 100 °C, respectively, matching the systems T-HKL-A and T-SKL-A. One notable difference to the lignin systems is the appearance of a second curing peak following the main curing peak, suggesting a more complex reaction in the mixtures containing lignin. In summary, the presence of accelerator promoted faster and more complete cross-linking. Epoxy systems T-A, T-HKL-A, and T-SKL-A were thus selected for the subsequent preparation of thermoset materials.

The herein prepared systems were also compared to bisphenol A diglycidyl ether resin (DGEBA) with regard to reactivity. Just as TGPh, neat DGEBA showed no curing exotherm without accelerator, whereas adding SKL initiated an exothermic reaction above ∼140 °C. Introducing the accelerator to DGEBA (DGEBA-A) promoted ring-opening reactions, and the DGEBA/SKL/accelerator mixture produced the highest enthalpy and lowest peak reaction temperature in this set. Nevertheless, the catalyzed DGEBA/SKL system cured at a higher peak temperature (∼160 °C) than the catalyzed TGPh systems (∼100 °C), indicating higher apparent reactivity of TGPh under otherwise similar conditions.

### Thermal Stability of the TGPh-Lignin Materials

Thermogravimetric analysis (TGA) was carried out to understand the thermal degradation behavior of the samples in oxidative atmosphere. The measurements were performed in duplicate (Figure 4).

**Figure 4:**
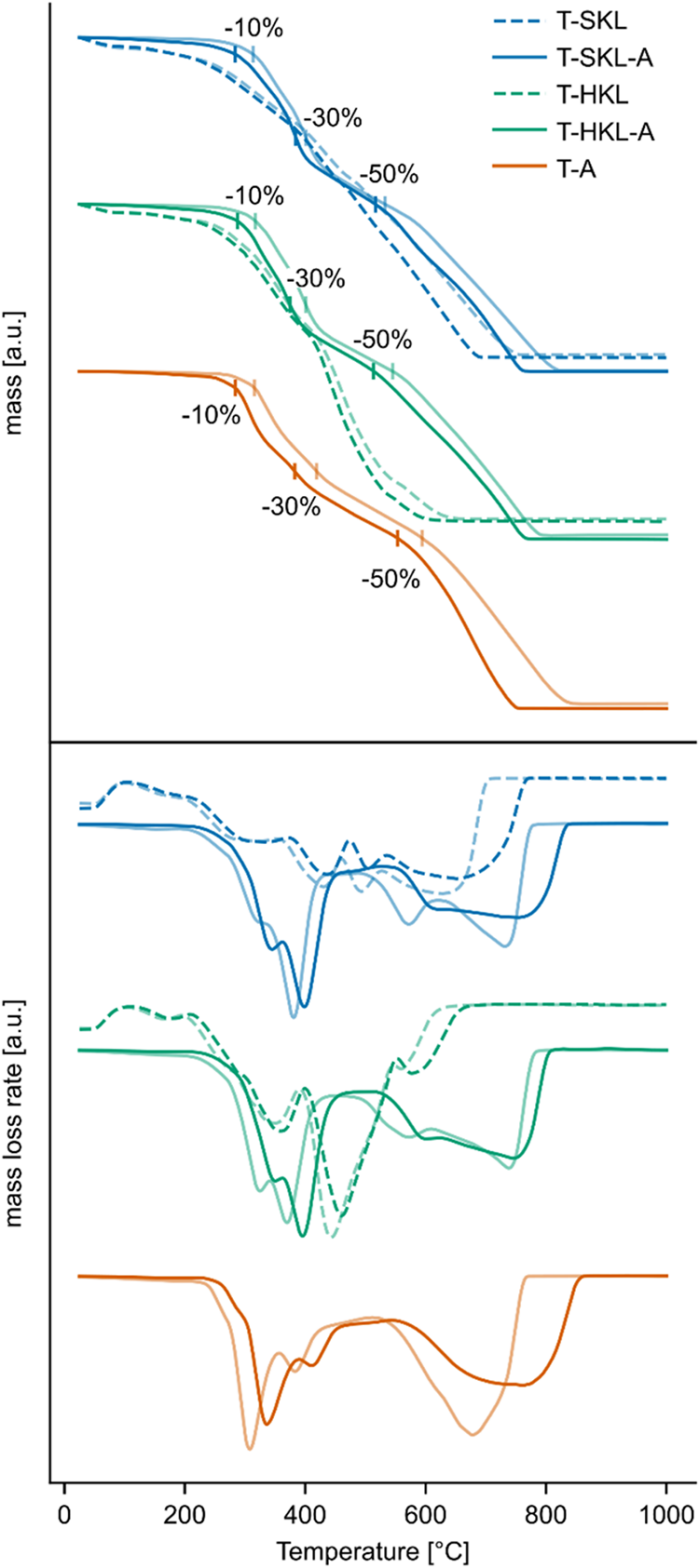
Mass loss (top) and derivative mass loss (bottom) determined by TGA analysis during heating from 25 to 1000 °C at 10 K/min under air. Light and strong colors represent two replicate measurements.

The TGA curves for the cured thermoset samples, including T-A, T-SKL-A, and T-HKL-A, displayed comparable degradation patterns, suggesting that the thermal degradation behavior was primarily governed by the TGPh component. All tested thermoset resins exhibited high thermal stability, as indicated by their *T*_10%_ > 280 °C and *T*_50%_ at temperatures of 500–600 °C. Obtained values for *T*_10%_ were very close for all three variants developed in the presence of accelerator, with temperatures between 280–320 °C. The presence of Kraft lignin in T-HKL-A and T-SKL-A led to a small decrease in thermal stability compared to T-A, particularly apparent at higher temperatures, as indicated by a decrease of the *T*_50%_ from 510–550 °C for the lignin thermosets to 550–600 °C for T-A. T-HKL-A and T-SKL-A performed similarly throughout the entire temperature range, indicated by their closely matching *T*_30%_ and *T*_50%_, and DTG curve (Figure 4 bottom). This is in contrast to measurements of neat KLs, which indicated that SKL exhibited lower thermal stability compared to HKL.

The mass loss in all thermoset samples followed a stepwise degradation pattern. The DTG thermograms showed that the mechanism of oxidative degradation is a combination of two major thermal events, with the first major DTG peak occurring at ∼300–400 °C, and the second at ∼600–800 °C. The first degradation event set in at slightly lower temperatures for T-A than for T-HKL-A and T-SKL-A, indicated by an earlier minimum in the DTG curve.

Surprisingly, while the residue was 4–6% for pure lignin, it decreased to values ∼ 0% in all tested thermoset samples. This difference can be attributed to the formation of stable char from neat lignin at high temperatures, which resists combustion. In contrast, the structure of the thermoset appears to hinder char formation during degradation, leading to more complete thermal decomposition.

To summarize, all tested thermoset resins exhibited high thermal stability matching the results of other studies.^37,38^ It has also been described that TGPh based resins may have a slower thermal decomposition compared to DGEBA-based epoxy resin cured under the same conditions.^37^ In the present study, the addition of 30% wt. of lignin slightly decreased the thermal stability. In previous studies, the addition of lignin had little or slightly negative effect on the thermal stability of epoxies,^28,30,51^ which is consistent with our findings.

### Thermomechanical Properties of TGPh-Lignin Thermosets

Dynamic mechanical analysis (DMA) was measured to understand the thermomechanical properties of the prepared biobased epoxy systems and the effect of KL on the epoxy network. Figure 5 depicts results for moduli on top and tan δ on the bottom, and Table 2 presents numeric results of 2 replicate measurements.

**Figure 5:**
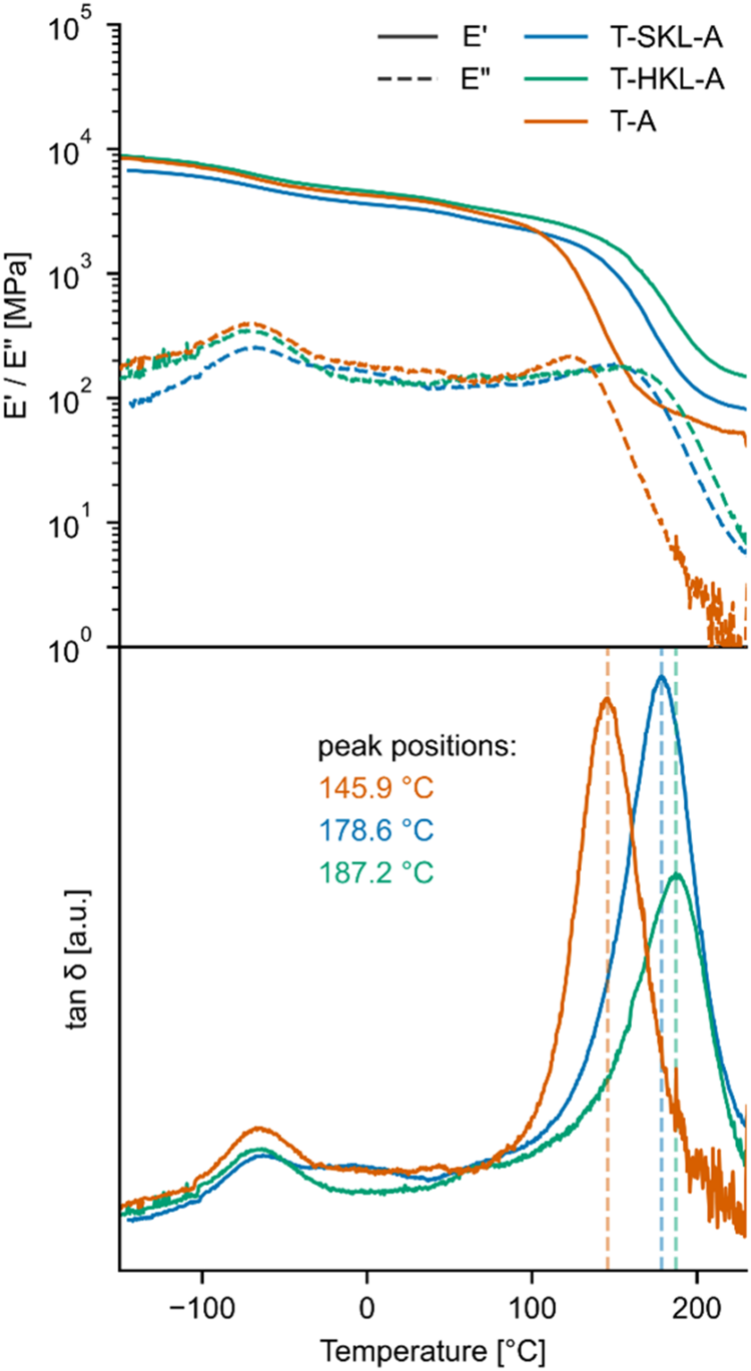
Dynamic mechanical analysis of prepared thermosets showing evolution with temperature of storage modulus *E*′ and loss modulus *E*″ (top) and tan δ (bottom).

**Table 2:** Summary of Results of Dynamic Mechanical Analysis

sample	*T*_α_ (°C)	*E*′@T_α_ + 80 °C (MPa)	*v* (mmol/cm^3^)	*E*′ at 25 °C (GPa)
T-A	145.9/152.6	52.8/-	4.2/-	3.95/4.13
T-SKL-A	178.6/177.7	80.6/76.7	6.1/5.8	3.37/3.16
T-HKL-A	187.2/178.2	143.6/108.0	10.7/8.2	4.19/3.95

The storage modulus (*E*′) at ambient laboratory temperature (estimated 20–25 °C) was found to be between 3.9 and 4.2 GPa for T-A and T-HKL-A, while T-SKL-A exhibited slightly lower values, of 3.4 and 3.2 GPa for two measurements, respectively. A peak at ∼50 °C in the loss modulus (*E*″) for all samples indicates subglass β-relaxation, which is associated with localized molecular motions within the polymer matrix. In the temperature range of 146 to 187 °C, peaks in the tan δ were observed, corresponding to the α transition of the polymer associated with the glass transition (*T*_g_). The *T*_g_ values were ∼146 °C/153 °C for TGPh, 178 °C/179 °C for T-SKL-A, and 187 °C/187 °C for T-HKL-A. This suggests that the addition of KL increased the glass transition values by about 30–40 °C.

Measurement of TGPh homopolymer at temperatures above 200 °C was challenging, due to significant softening of the material in the DMA device, rendering the determination of its cross-link density difficult. The cross-link density (*v*) was calculated at *E*′ = *T*_α_ + 80 °C, in the rubbery region. Generally, *v* ranged from 4 to 11 mmol cm^–3^. Samples containing lignin had higher cross-link densities than T-A samples, with the highest values observed in HKL samples (10.65 mmol cm^–3^/8.15 mmol cm^–3^), likely due to the lower molecular weight of HKL compared to SKL. The high cross-linking density and stiffness of the T-HKL-A is also reflected in the lower amplitude of the tan δ peak.

Excellent thermomechanical properties of TGPh cured with hardeners have been described in the literature (Table 3).^32,35,37^ Several articles have reported higher thermomechanical properties of TGPh compared to DGEBA resin, cured under the same conditions.^37,35,38^ This effect may be related to its trifunctional character. Herein, for the first time, the thermomechanical properties of homopolymerized TGPh without hardeners are reported. Obtained properties of homopolymerized TGPh (T-A) with *T*_g_ of ∼150 °C is in the range for high performance materials suitable in structural applications in the automotive and construction industries. The performance is comparable to properties of TGPh cured with hardeners as well as to the performance of classical epoxy resins.

**Table 3:** Comparison of Glass Transition Temperature and Tensile Properties of Homopolymerized TGPh in This Study with TGPh-Based Epoxy from Other Articles

system	*T*_g_ (°C) by DMA	tensile strength (MPa)	tensile modulus (GPa)	elongation at break (%)	refs
TGPh homopolymer (T-A)	146–153	51	2.2	3.2	this study
TGPh + aromatic amine	260	67	1.8	—	Dyer et al.^37^
TGPh + cyclic/other anhydrides	86–163	21–62	1.2–1.6	2.6–6.6	Dinu et al.^38^
TGPh + 4-aminophenyl disulfide	197	92	1.2–1.5	5.0	Genua et al.^32^
TGPh + Jeffamine	73	28	2.2	3.2	Santiago et al.^35^

Other studies came to the conclusion that the addition of lignin to epoxy resins may both enhance or diminish cross-link density as well as the glass transition temperature.^22,23,28,30^ The herein reported resins show a clear enhancement of the glass transition temperature with addition of lignin. It can be speculated that the effect of plasticization or enhancement of thermomechanical properties may be related to the compatibility of lignin and epoxy resin and/or to the extent to which lignin participates in the cross-linking of the resin. Moreover, the added quantity was reported to play a significant role as well.^16^ One study has attributed the decrease of cross-link density and *T*_g_ to the higher molecular weight of lignin between cross-links.^51^ This relationship between molar mass of the segments between cross-links may be a potential explanation for the lower cross-linking density of samples T-SKL-A (*M*_w_SKL_ = 14,929 g/mol) compared to T-HKL-A (*M*_w_HKL_ = 5494 g/mol).

### Tensile and Hardness Properties of TGPh-Lignin Thermosets

Tensile testing was conducted to evaluate the macroscopic mechanical properties of the prepared thermoset materials. Samples prepared from TGPh with accelerator (T-A) had a mean tensile strength of ∼50 MPa, with values ranging from 30–75 MPa (Figure 6a), consistent with literature values, which reported comparable strength for similar TGPh based resins.^37^ For samples containing 30% wt. KL, the tensile strength decreased significantly, with T-SKL-A, and T-HKL-A exhibiting mean tensile strengths of 20 and 16 MPa, respectively. Moreover, while mean elongation at break for T-A was 3.2%, this value decreased to around 1% for the lignin-containing samples (Figure 6d). The lower tensile strength and reduced strain at break in lignin-containing samples indicate increased brittleness. Contrary to these observations, the stiffness of the material was not significantly affected by the addition of lignin (Figure 6c). This is reflected by a comparable tensile modulus with values ranging between 2150–2260 MPa for all tested variants.

**Figure 6:**
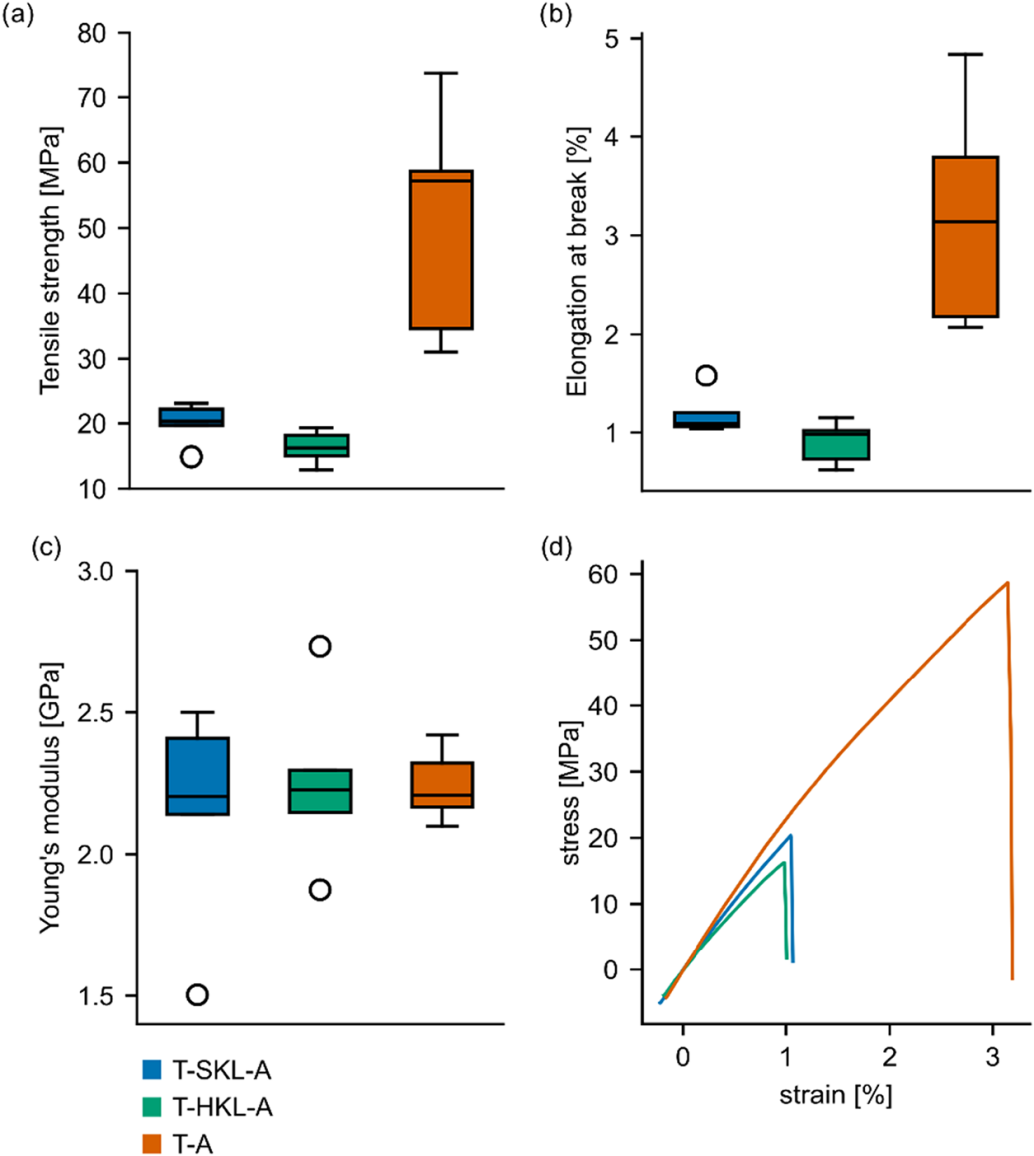
Results of tensile testing on cured epoxy resins showing ultimate tensile strength (a), elongation at break (b), Young’s modulus (c), and exemplary stress–strain curves (d).

Figure 6d shows representative stress–strain curves for the different sample variants. The curves clearly illustrate the differences in mechanical behavior, with T-A homopolymer showing higher tensile strength and elongation compared to the lignin-containing thermosets. The area under the stress–strain curves, which represents the toughness of the material, was significantly larger for T-A compared to T-HKL-A and T-SKL-A. This signifies that T-A can absorb more energy before failure, meaning increased toughness, again highlighting the comparable brittleness of the lignin-containing samples.

The mechanical properties of the homopolymerized TGPh, with a tensile strength of ∼50 MPa, 3% strain at break, and a tensile modulus of 2.2 GPa, are comparable to those reported in the literature for TGPh cured with various hardeners (Table 3). For instance, TGPh cured with aromatic amine cross-linker achieved a tensile strength of 67 MPa and a tensile modulus of 1.79 GPa.^37^ Additionally, TGPh cured with various anhydrides showed tensile strengths ranging from 21 to 62 MPa, elongations from 2.6% to 6.6%, and stiffness values between 1.2 and 1.6 GPa.^38^ TGPh cross-linked with 4-aminophenyl disulfide achieved a tensile strength of 92 ± 6 MPa at 5% strain.^32^ Another study reported TGPh cured with Jeffamine exhibiting a tensile strength of 28 MPa at 3.2% elongation.^35^ The observed decrease in mechanical properties in lignin-containing samples is partly consistent with the other reports from the scientific literature. Similarly as described above for the glass transition, the addition of lignin to epoxy resins has also been reported to both enhance or diminish mechanical properties, depending on the specific formulation and processing conditions.^22,23,28,51^ For instance, replacing the amine hardener in DGEBA epoxy with 10% carboxylated lignin increased the Young’s modulus, tensile strength, elongation at break, and consequently the toughness.^51^ Similarly, when organosolv lignin was mixed into DGEBA epoxy cured with Jeffamine, higher values for tensile strength and modulus were initially obtained. However, beyond a certain loading, the tensile strength decreased while the modulus remained high. The authors explained the more brittle nature of the materials at higher lignin loadings by the disruption of the epoxy network caused by the bulky lignin particles.^23^ Some other studies have also reported decreases in mechanical properties with the addition of lignin. In systems involving a diglycidyl ether, i.e., the resorcinol diglycidyl ether, when cured with 30% lignin, values of approximately 15 MPa tensile strength, 1 GPa tensile modulus, and 3% elongation at break were observed, which were lower than those of the comparison samples, matching our observations.^28^ In another study, replacing pyrogallol with lignin in the curing of glycerol epoxy also resulted in a decrease of the tensile strength and Young’s modulus.^22^

The Shore D hardness values (Table 4) for all the resins ranged between 80 and 85, classifying them as extra-hard materials, a level of hardness which is typical for epoxy resins. Previous studies have moreover reported high hardness values for TGPh-based resins compared to other epoxy resins cured under similar conditions.^35^

**Table 4:** Shore D-Hardness Values of Prepared Thermosets

	shore D hardness		
variant	mean	SD_within_	SD_between_
T-A	81.4	2.6	3.2
T-HKL-A	83.9	3.4	2.1
T-SKL-A	82.1	2.4	2.3

The addition of 30% wt. HKL or SKL did not significantly alter the hardness values. Hardness in thermosetting resins is associated with mechanical properties such as strength and stiffness, however, the relationship is complex and not necessarily proportional.^52^ The effect of lignin on the hardness and stiffness of the epoxy matrix has been documented in the literature for an epoxidized plant oil matrix: When lignin acted as a plasticizer, it tended to lower both the modulus and hardness.^30^ Conversely, when lignin increased the stiffness of the material, it also enhanced the hardness.^30^ In our study, the addition of 30% wt. lignin did not lead to a significant change in hardness, suggesting that the lignin did not act as a plasticizer in this context. One study has recently reported high hardness values exceeding 90 on the shore D scale when 30% lignin was added to biobased epoxy resins.^28^

### Microstructural Analysis

Scanning electron microscopy of fracture surfaces was carried out to investigate the presence of defects, inhomogeneities, or phase separation in the samples. Two specimens of each of the three tested variants were examined. Fracture surfaces of T-HKL-A and T-SKL-A (Figure 7a,b) displayed noticeable pores and holes distributed over the surface. Conversely, the fracture surface of T-A was smooth, without evident defects (Figure 7c). The pores in the lignin-containing samples may stem either from air inclusions during formulation preparation, or from gases released during curing, such as water vapor from absorbed moisture.^53^ A potential explanation is the much-elevated viscosity of T-HKL and T-SKL mixtures, compared to the pure TGPh resin: In the literature it has been shown that vacuum degassing is limited to low-viscosity resins.^53^ Although vacuum degassing was not used in this study, the elevated viscosity of the lignin formulations likely hindered the escape of entrained air and cure-released volatiles. Because these air inclusions cause a strong reduction of the actual surface area compared to the apparent surface area, it can be assumed that the measured mechanical properties are an underestimation of the actual material performance.

**Figure 7:**
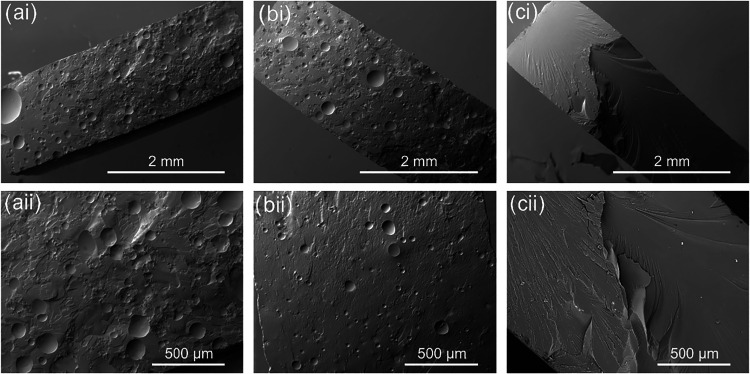
Scanning electron microscopy imaging of fracture surfaces of tensile specimens prepared from thermosets T-HKL-A (a), T-SKL-A (b), and T-A (c) shown at 40× (i) and 100× (ii) magnification.

### Gel Content Measurement

A gel content analysis was carried out to determine the extent of cross-linking in the thermoset materials. A high GC% indicates a well-cross-linked network, which is essential for the mechanical integrity and chemical resistance of cured thermosetting resins. The gel content of TGPh-based epoxy resins, with and without KL, was evaluated. Results are presented in Table 5.

**Table 5:** Gel Content (GC%) of Prepared Thermosets

	toluene		THF		acetone		water	
variant	mean	s	mean	s	mean	s	mean	s
**T-A**	100.1	0.3	99.8	1.1	98.2	1.9	99.4	0.5
**T-HKL-A**	99.9	1	101.1	1.8	97.7	1.2	99.5	0.3
**T-SKL-A**	99.2	0.6	100.0	2.5	98.8	1.0	99.4	0.3

The results in this study showed that all samples, including those cured with hardwood and softwood KL, retained masses >97%–100% when immersed in toluene, tetrahydrofuran, acetone, or water for 3 days. Samples immersed in THF show very high values–likely an overestimation related to the presence of nonvolatile stabilizer after drying. The high gel content of all samples suggests that the curing process was effective, leading to nearly complete cross-linking of the epoxy network. The incorporation of KL did not adversely affect the gel content, indicating that lignin was well integrated into the TGPh matrix and participated effectively in the curing process.

The high gel content observed in all samples in this study is consistent with the findings from FTIR and DSC analyses, which indicated an interaction between lignin and the epoxy resin. The phenolic OH groups in lignin likely played a crucial role in the epoxy ring-opening reactions, contributing to the formation of a cross-linked network. Moreover, the gel content results align with the mechanical testing outcomes, where the stiffness of the lignin-containing samples was maintained despite a decrease in tensile strength. This suggests that the cross-linked network formed in the presence of lignin was sufficiently dense to provide structural integrity.

Along with the gel content, the swelling ratio was measured as the mass of the swelled sample divided by the mass of the oven-dried sample. The swelling ratio was 100–102% for all three sample types when immersed in acetone or THF, which is related to no or very low absorption of solvent. For samples immersed in water, the swelling ratio was at 102% for T-A, but increased to 107% and 106% for T-HKL-A and T-SKL-A, respectively, indicating the increased hydrophilicity of samples containing lignin.

## Conclusion and Outlook

This study demonstrates the formulation of epoxy resins using potentially biobased phloroglucinol triglycidyl ether (TGPh) with and without the addition of unmodified KL. The incorporation of 30% wt. lignin into the TGPh matrix increased the glass transition values and the cross-link density. While the presence of lignin decreased the tensile strength of the materials, the stiffness was maintained with a tensile modulus ∼2.2 GPa. The interaction of lignin with the epoxy resins, as indicated by DSC and FTIR, suggests that lignin acts as a cohardener, enhancing the thermomechanical properties of the resins. The high gel content >99% is proof of the successful incorporation of KL into the resin matrix.

Regarding the comparative performance of SKL and HKL, the results suggest a slightly increased reactivity of SKL over HKL. In DMA measurements, a somewhat higher cross-linking density, higher *T*_g_ values, and a lower tan δ peak amplitude for HKL over SKL were found, which can potentially be attributed to its lower molar mass and resulting denser network. In terms of mechanical properties and hardness, a similar performance of thermosets incorporating HKL and SKL was found.

Future research should focus on mechanistic studies, based on spectroscopic methods such as NMR, to gain a deeper structural understanding of the interactions between lignin and the epoxy matrix. Additionally, different types of lignins and varying concentrations need to be studied to determine which lignin works best and why, as well as how to increase the compatibility and reactivity of lignin with the epoxy matrix. This understanding will help more effectively incorporate KL into thermosetting resins.

Besides investigating the addition of unmodified KL to a biobased epoxy resin, this is the first study to investigate the homopolymerization of TGPh, revealing a glass transition temperature of ∼146 °C, tensile strength ∼51 MPa, and tensile modulus of ∼2.2 GPa.

## Supplementary Material



## Notes

(1) Size exclusion chromatogram of KL (Figure S1); (2) Molar mass results of lignin (Table S1); (3) Results of ^31^P NMR (Table S2); (4) Various FT-IR spectra (Figures S2–S4) (PDF)
