# Supplementary material for: High Glass Transition Epoxy Resins from Biobased Phloroglucinol and Unmodified Kraft Lignin
Source: ACS Omega. 2025 Nov 4;10(45):54236–48. doi: 10.1021/acsomega.5c06542 (PMC12631697; doi:10.1021/acsomega.5c06542)
Supplement: Supplementary file 1 [file ao5c06542_si_001.pdf]

Supporting Information

# High glass transition epoxy resins from biobased phloroglucinol and unmodified Kraft lignin

Jan Janesch*[1,2], Roxana Dinu[5], Thomas Rosenau[2], Antje Potthast[2], Wolfgang Gindl-Altmutter[1,3], Stefan Grasböck[2], Irina Sulaeva[4], Alice Mija*[5]

1 BOKU University, Institute of Wood Technology and Renewable Materials, Department of Natural Sciences and Sustainable Resources, Konrad-Lorenz-Straße 24, 3430 Tulln, Austria.

2 BOKU University, Institute of Chemistry of Renewable Resources, Department of Natural Sciences and Sustainable Resources, Konrad-Lorenz-Straße 24, 3430 Tulln, Austria.

3 Wood K plus - Competence Centre for Wood Composites & Wood Chemistry, Kompetenzzentrum Holz GmbH, Altenberger Straße 69, 4040 Linz, Austria

4 BOKU University, Core Facility Analysis of Lignocellulosics (ALICE), Konrad Lorenz-Strasse 24, 3430 Tulln, Austria

5 Côte d'Azur University, Institute of Chemistry of Nice, UMR CNRS 7272, 06108 Nice CEDEX 2, France

*Corresponding authors: jan.janesch@boku.ac.at, alice.mija@univ-cotedazur.fr

Contents:

Number of pages: 4

Number of tables: 2

Number of figures: 4

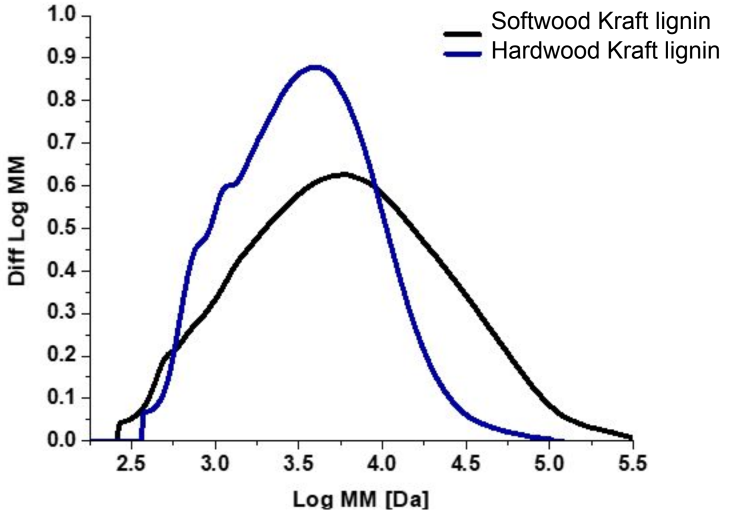


Figure S1: Size exclusion chromatogram of Kraft lignin.

*Table S1: Molar mass results of lignins*

| Sample | Mn (Da) | Mw (Da) | Mz (Da) | Đ |
|---|---|---|---|---|
| Indulin AT | 2622 | 14929 | 63311 | 5.693 |
| Suzano | 2178 | 5494 | 14559 | 2.522 |

*Table S2: Results of functional group quantification conducted with $^{31}P$ NMR.*

| Sample | OH aliph. (mmol/g) | OH phen. (mmol/g) | COOH (mmol/g) |
|---|---|---|---|
| Indulin AT | 2.37 | 3.89 | 0.49 |
| Suzano | 1.16 | 4.26 | 0.33 |

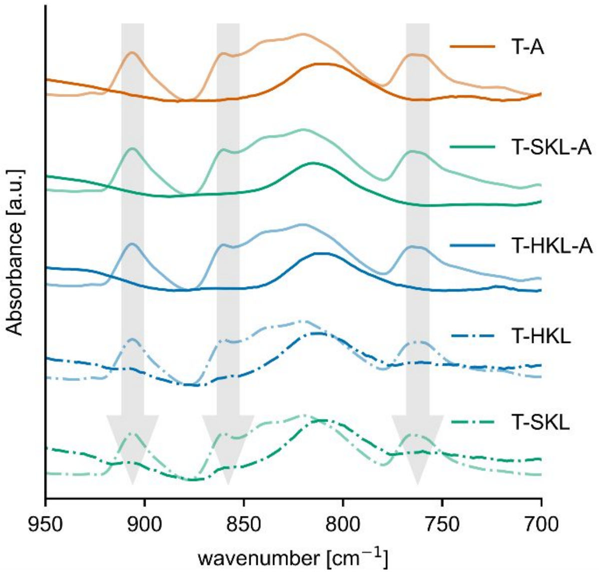


Figure S2: FTIR spectra obtained from fresh unreacted mixtures (light color) and samples after post-curing in the oven at 200°C (intense color).

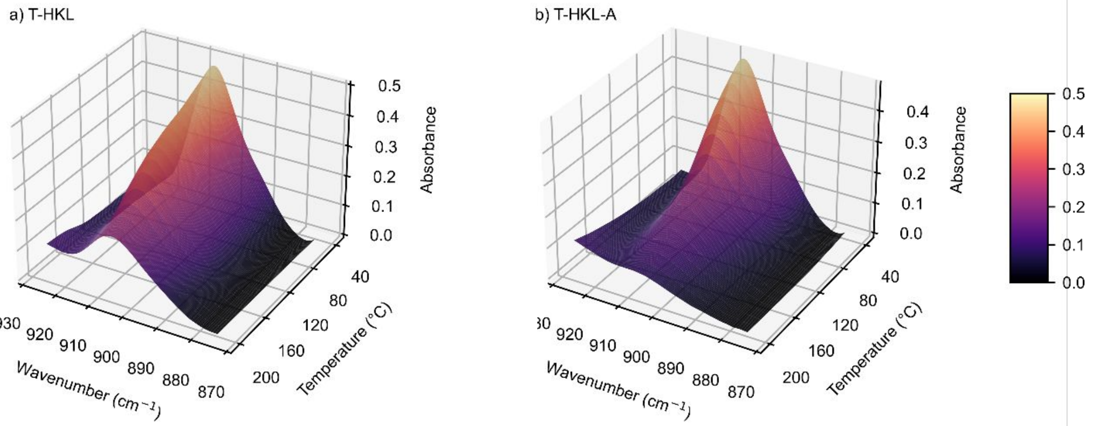


Figure S3: Evolution of epoxy peak intensity in thermally-resolved FTIR using mixtures of TGPh and hardwood Kraft lignin without (a) and with (b) accelerator.

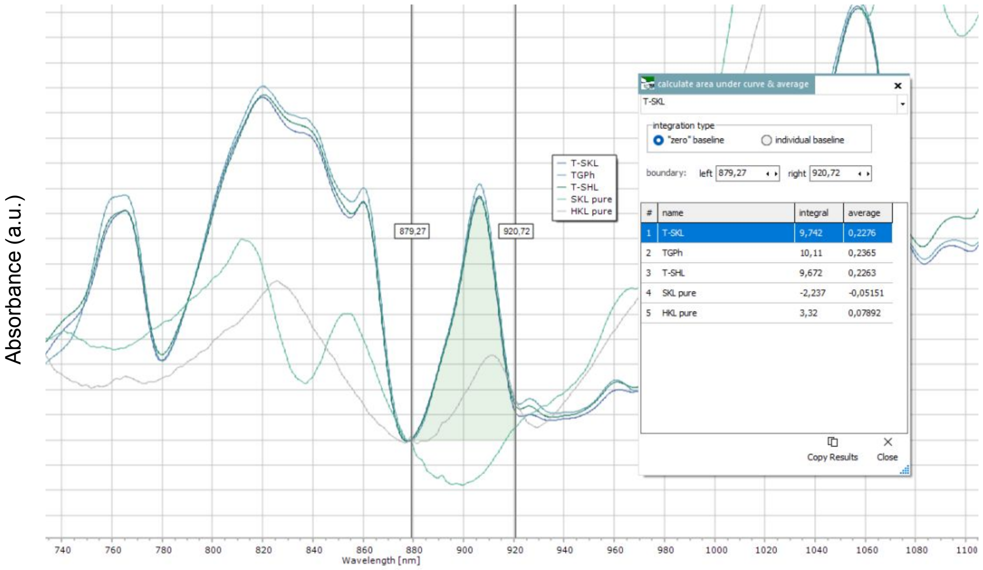


Figure S4: FTIR spectrum of pure Kraft lignins and mixtures of TGPh and Kraft lignins, showing the overlap of the epoxy vibration at 912 $cm^{-1}$ with the integrated area.
